# Exploring the feasibility of smartglass facilitated remote supervision in the emergency department: A simulation study

**DOI:** 10.1111/1742-6723.14142

**Published:** 2022-12-13

**Authors:** Arthur McTavish, Peter Larsen, Alice Rogan, Emma Carlin, Matthew Lynch, Brad Peckler

**Affiliations:** ^1^ Department of Surgery and Anaesthesia University of Otago Wellington New Zealand; ^2^ Department of Emergency Medicine Capital and Coast District Health Board Wellington New Zealand; ^3^ Clinical Lead Simulation and Skills Centre Capital and Coast District Health Board Wellington New Zealand

**Keywords:** Google glasses, simulation, telemedicine

## Abstract

**Objective:**

Smartglasses are a wearable computer technology that has potential to facilitate remote supervision to junior doctors working in different clinical settings. The present study aimed to explore the feasibility of smartglass technology to enable remote supervision of junior clinicians by senior clinicians during emergency simulation scenarios.

**Methods:**

This was a feasibility simulation study using high‐fidelity mannequins and standardised patients. Trainee interns (TIs) and supervising clinicians (SCs) were invited to participate in two scenarios: a trauma case and a stroke case. There was a pre‐sim questionnaire. The TI wore the smartglasses in a simulated ED bay and performed patient assessment and management. Remote supervision was provided by the SC via a livestream on a remote computer. Upon completion, participants completed a survey regarding their experience comprising of Likert scale and free‐text questions.

**Results:**

Fifteen TIs and 19 SCs participated. In general feedback from TIs and SCs was positive. TIs were able to identify and treat the key diagnostic problems set during the scenarios. Free‐text survey responses provided specific feedback about what did and did not work when using the glasses.

**Conclusion:**

The present study demonstrates that smartglasses facilitated remote assistance has promise as an emergent technology and warrants further investigation in simulated and non‐simulated environments.

## Introduction

Smartglasses are a wearable computer technology that may have a use in enabling clinicians to supervise other clinician's practice remotely. Smartglasses could enable ‘real‐time’ clinical supervision and support by adding functionality found in devices such as smartphones and tablets to eyeglasses. The present study aimed to explore the feasibility of smartglass technology to enable remote supervision of trainee interns by senior specialists or supervising clinicians (SCs) during emergency simulation scenarios.

## Methods

**Figure 1:**
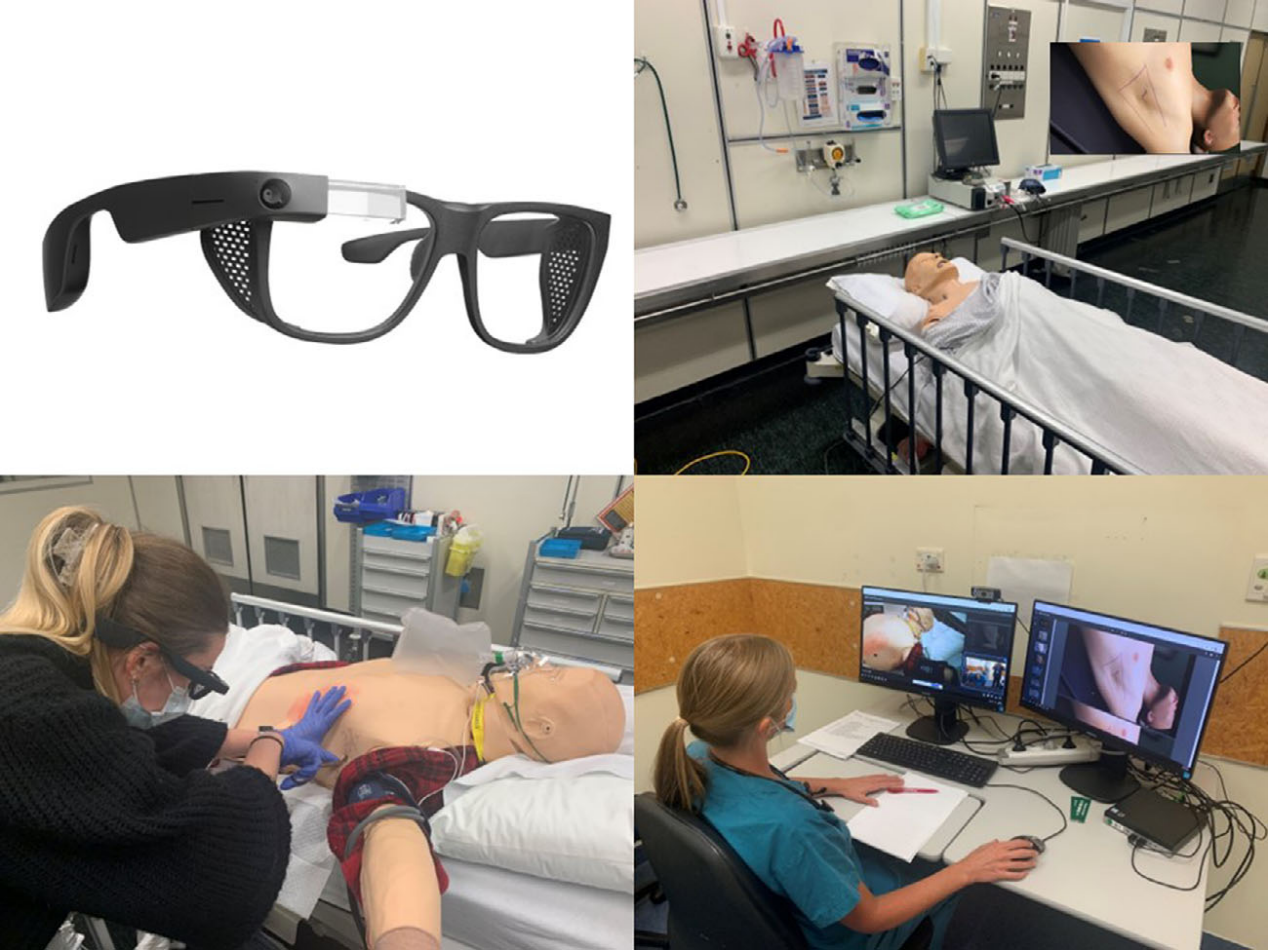
From top left clockwise: Google Glasses Enterprise Edition 2, view of smartglasses in use, remote clinician setup, trainee intern performing finger thoracostomy wearing smartglasses.

This was a proof‐of‐concept design and feasibility simulation study (Fig. 1) using high‐fidelity manikins and standardised patients conducted within Wellington Regional Hospital Simulation Department in collaboration with the University of Otago, Wellington.

Trainee interns (TIs), 6th year medical students, were invited to volunteer to participate within the study via email sent to all TIs at the University of Otago, Wellington. Emergency specialists, emergency senior registrars and neurology specialist clinicians were selected to provide remote supervision via the smartglasses. We called this group SCs.

TIs participated in two simulations: a major trauma scenario and a FAST‐positive stroke presentation designed in an ED setting. The TI was tasked to evaluate and manage each scenario with support from the SC via the smartglasses.

In the trauma case, the TI was tasked to perform a primary survey, recognise a tension pneumothorax and perform a finger thoracostomy and recognise and treat intra‐abdominal bleeding on a 35‐year‐old male post‐motor vehicle accident. In the stroke case, the TI was tasked to recognise and treat hypoglycaemia and recognise the need for thrombolysis in a 65‐year‐old male or female with left middle cerebral artery syndrome. The trauma case used a high‐fidelity mannequin, whereas the stroke used an actor/actress. These cases are summarised in Table S1.

The trauma case evaluated in the emergency trauma situation if the smartglasses are an effective aid in facilitating conduction of a primary survey, recognition of a deteriorating patient and performing a procedure, and are helpful in making time conscious decision making. The stroke case evaluated if it can be used in a context that is heavily dependent on physical examination and communication of findings to a senior to aid in clinical decision‐making.

Upon completion of each simulation case, a structured debriefing was conducted with the participants. Participants were then asked to complete an online survey regarding their experience using the technology.

The TI used the Glass Enterprise Edition 2. For videoconferencing, Google Meet was used. The smartglasses were connected to a mobile hotspot for internet connection as they were incompatible with hospital Wi‐Fi. The SC used a computer with a second monitor available. The SC initially used a MacBook Air with a display using department Wi‐Fi for our study, but midway through the study, this was changed to a department desktop terminal with zoom capability and two displays and ethernet connection to the internet. The speakers used were the internal ones of the MacBook and later the desktop display. The microphone used by the SC was the microphone of the MacBook Air and then later that of a peripheral webcam.

No PPE eye protection was worn in these scenarios.

## Results

### Recruitment and participant characteristics

**Figure 2:**
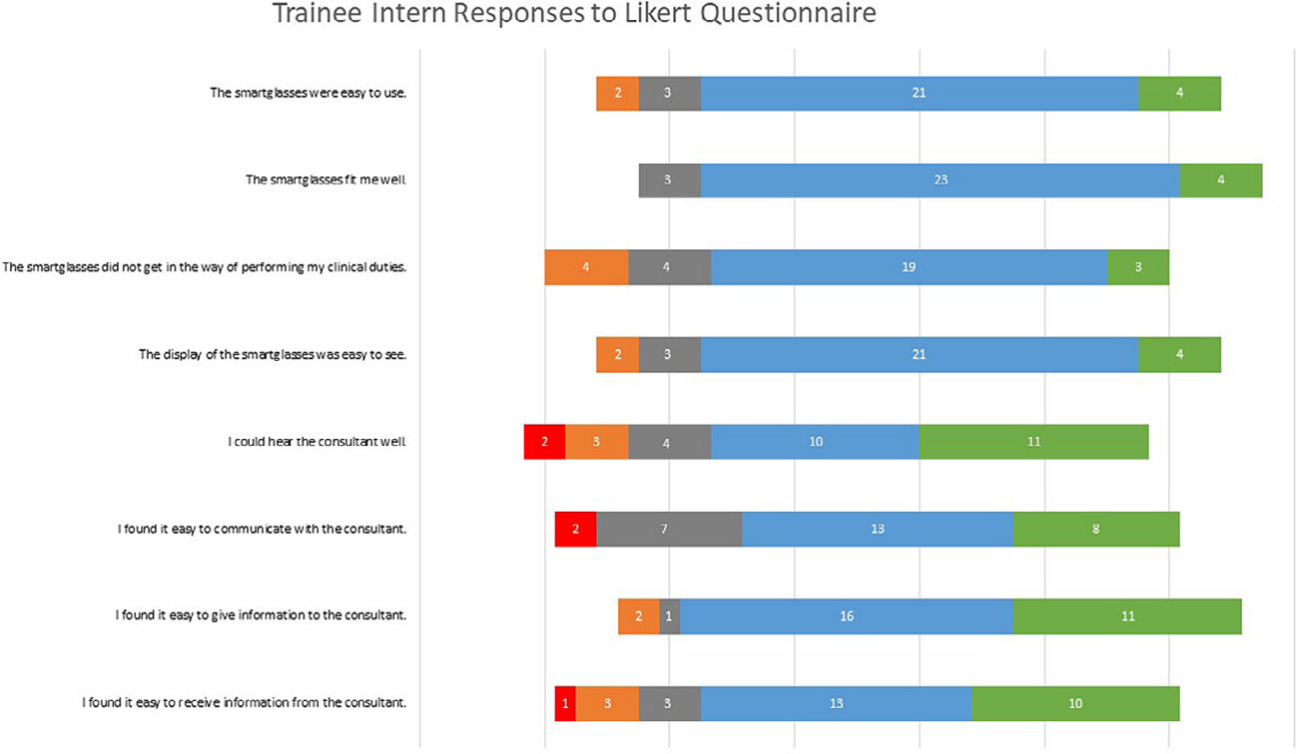
Trainee intern responses to Likert questionnaires. Responses from both the trauma and stroke presentations are pooled. (), Strongly disagree; (), disagree; (), neutral; (), agree and (), strongly agree.

We recruited 15 TIs and 19 SCs for our study, resulting in a total of 34 participants. A summary diagram of Likert survey responses for TIs is presented in Figure 2 and for SCs in Figure S1. All TIs identified the tension pneumothorax and proceeded to finger thoracostomy. About 13/15 TIs identified the intra‐abdominal bleed in the trauma simulation. All TIs identified the hypoglycaemia and need for thrombolysis within the stroke simulation.

Written feedback was obtained in free‐text responses to the questions:

- TIs were not familiar with volume control, and sometimes audio quality was the major limiting factor in the performance of the glasses.
- It was helpful for other scenario participants, for example, the simulated ED nurse to hear the TI, but this was not always reliable.
- The field of view was too high which limited the ability for the clinician to see the procedural work.
- SCs reported that their situational awareness was reduced by the smartglasses leading them to become more task focused with their approach to the patient.
- It was noted that closed‐loop communication with call‐out of examination findings was a useful tool to facilitate communication.
- Some technical difficulties were reported including momentary drop out of audio‐visual feeds requiring repeated communication at times.
- TI's reported that the glasses were appropriately sized and weighted for clinical use.

Eight TIs regularly wore eyeglasses. Of these, five decided to conduct the simulation without wearing them, whereas one wore the smartglasses over their eyeglasses during the study. Two TIs wore contact lenses for the study.

## Discussion

This was an exploratory feasibility and proof‐of‐concept study that investigated the application of smartglasses to facilitate remote supervision between a junior doctor and a supervising senior clinician. Although there were some technical limitations, in general use of smartglasses was viewed favourably by participants and all TIs were able to successfully assess and manage the patient via remote supervision. We believe suitable proof of concept has been achieved to warrant further research of this technology.

There were some issues that were noted in the written feedback regarding internet connections and field of view. There may be some simple protocols and procedures that could be put in place to address some of the aforementioned issues in order to optimise the performance of smartglasses in the clinical environment. Sporadic issues with audio quality were likely because of poor internet connection, and thus a reliable internet connection is essential to reliable communication. A simulation education session prior to use in the clinical environment would be useful. A face shield could feasibly be worn over the glasses to facilitate COVID precautions.

Limitations of the study include the evaluation of the smartglasses in a single centre, small sample size and the simulated context. Ideally, a larger confirmatory study would be required. The SCs were not blinded to the need for a finger thoracostomy because of familiarisation with clinical information available for display to the TI. The small sample size and the in‐person debriefing followed by a survey means that there may be a degree of bias in the responses as they may feel pressure to respond in a certain way as they may feel that as their responses are potentially identifiable by the examiners. We decided that for our study, an in‐person debriefing was best practice following a simulation event.

## Supporting information


**Figure S1.** Clinician responses to Likert questionnaires. Responses from both the trauma and stroke presentations are pooled.

**Table S1.** Summary table of trauma and stroke simulations used in the present study.

## Data Availability

The data that support the findings of this study are available from the corresponding author upon reasonable request.

