# Supplementary figures and images for: Exploring the feasibility of smartglass facilitated remote supervision in the emergency department: A simulation study
Source: Emerg Med Australas. 2022 Dec 13;35(1):170–2. doi: 10.1111/1742-6723.14142 (PMC10107718; doi:10.1111/1742-6723.14142)

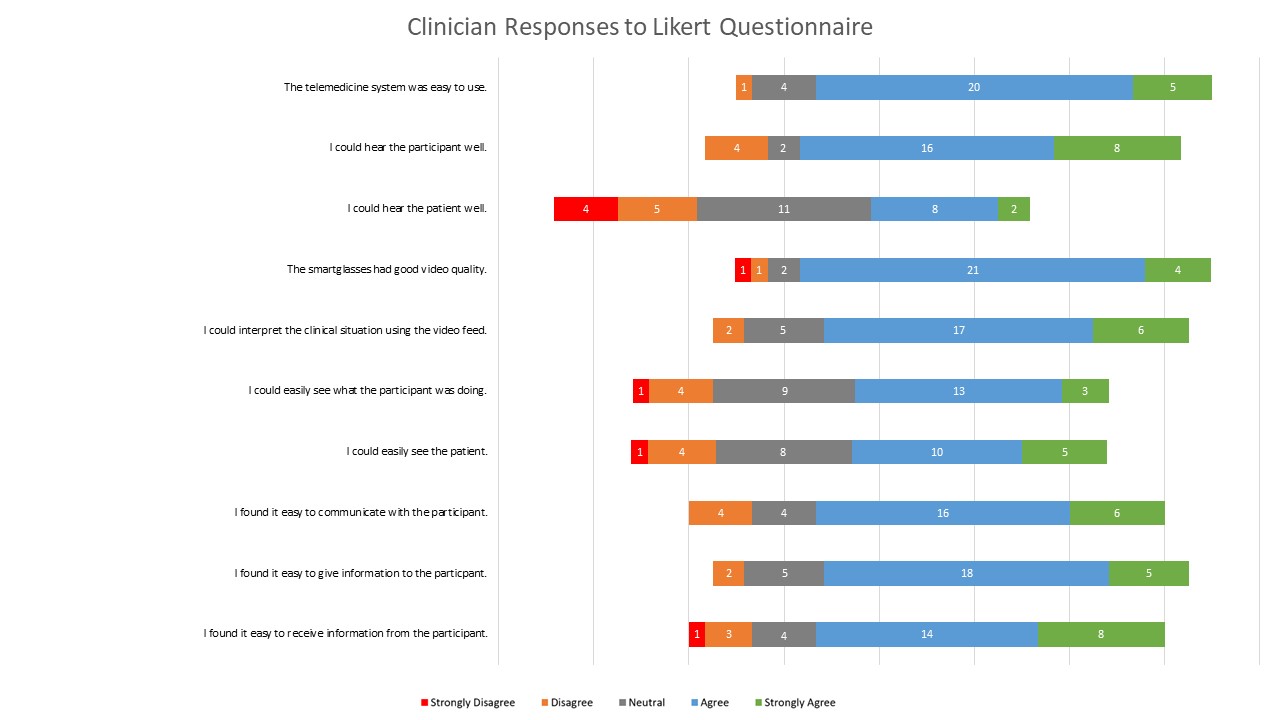

Supplement: Supplementary file 1 — Figure S1. Clinician responses to Likert questionnaires. Responses from both the trauma and stroke presentations are pooled. [file EMM-35-170-s001.JPG]

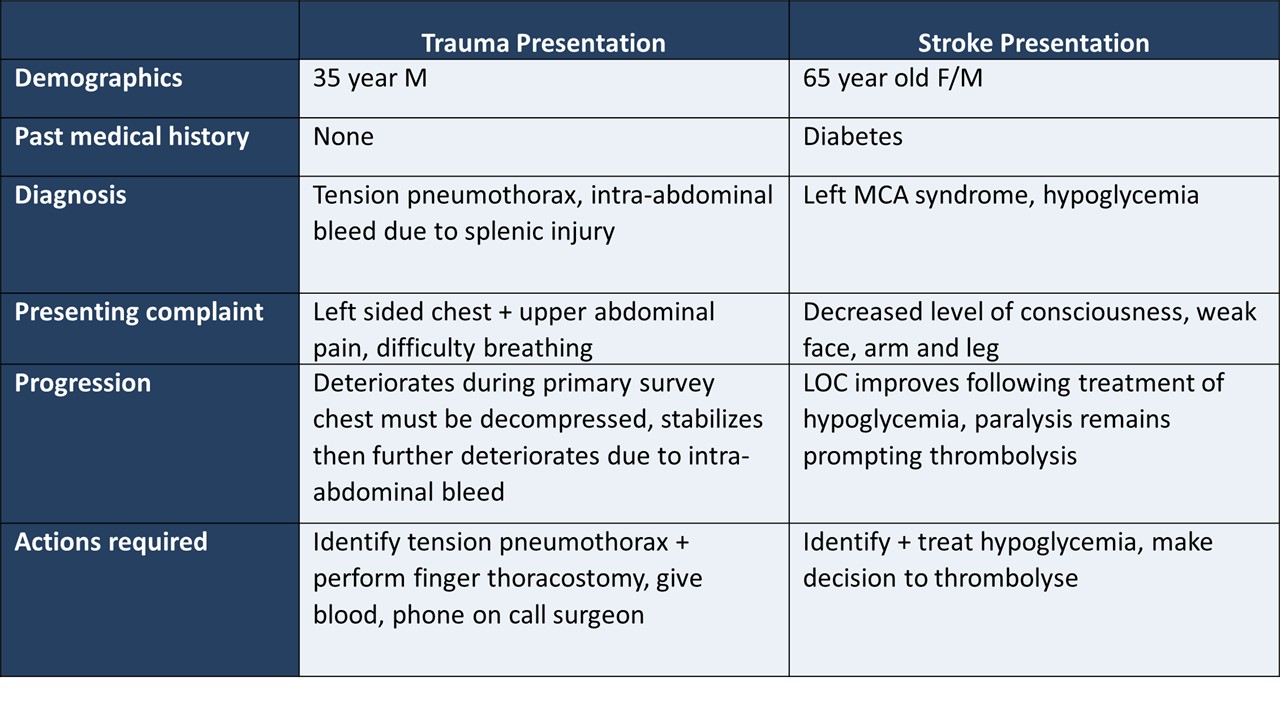

Supplement: Supplementary file 2 — Table S1. Summary table of trauma and stroke simulations used in the present study. [file EMM-35-170-s002.JPG]
